# EEG-based stress classification using time-domain features and segmentation techniques

**DOI:** 10.1038/s41598-026-50857-9

**Published:** 2026-05-28

**Authors:** Usman Rauf, Anfal Zahid, Amina Qadeer, Adeel Zafar, Shehrayar Khan, Sanay Muhammad Umer Saeed

**Affiliations:** 1 Computer Engineering Department, University of Engineering and Technology, Taxila, Pakistan; 2 Computer Engineering Department, Halmstad University, Halmstad, Sweden; 3 Computer Engineering Department, HITEC University, Taxila, Pakistan

**Keywords:** Time Domain analysis, Feature selection, Electroencephalography (EEG), Perceived stress, Segmentation technique, Computational biology and bioinformatics, Engineering, Mathematics and computing, Neuroscience

## Abstract

Stress has been recognized as a significant global health issue, affecting the majority of the population. Rapid and accurate detection of stress is critical for stress treatment. A considerable part of prior work has focused on classifying electroencephalography signals to enable preliminary detection of stress. Previous work has demonstrated considerable success in stress classification/detection using electroencephalography signals. The proposed work aims to classify human stress using electroencephalography signals to enable early intervention. In this research, we have worked with a dataset comprising nearly 211 individuals and proposed a method based on time-domain analysis. Segmentation techniques are used to analyze stress from EEG signals. Both overlapping and non-overlapping methods are employed in this research work. The EEG signals last approximately 480 seconds. We have used the Perceived Stress Questionnaire (PSQ) for the labeled classes of ‘stressed’ and ‘non-stressed’. Different classifiers have been employed to distinguish between stressed and non-stressed classes. Our proposed method achieved an accuracy of 96.32% using a K-nearest neighbors classifier with a non-overlapping segmentation technique.

## Introduction

The reaction a person experiences, whether psychological or physiological, may be considered stress. Stress has become a major global health concern, affecting a substantial portion of the population. Using data from the Gallup World Poll, studies have shown that 35.1% of the global population experiences stress^1^. Women have been reported to show a higher prevalence of stress than men^2^. In more than 20 countries, nearly half of the population experiences symptoms of emotional stress, while younger individuals show a notable decline in physical, mental, psychological, and physiological well-being compared with older adults^3^. According to the World Health Organization, nearly 15% of working adults are affected by stress-related problems.

Stress-related disorders are estimated to result in the loss of 12 billion workdays annually^4^. Stress is typically triggered by specific internal or external stimuli and has been associated with several adverse health outcomes, including cardiovascular disease^5^, immune system dysfunction^6^, diabetes mellitus^7^, and stroke^8^. Therefore, accurate assessment of stress is essential for timely intervention and effective management^9^. Traditionally, stress has been measured using interviews, questionnaires, and psychological sessions conducted by trained professionals. Several standardized instruments have been developed for this purpose, usually based on rating scales that quantify perceived stress levels^10^. Common examples include the Perceived Stress Questionnaire (PSQ)^11^, the Perceived Stress Scale (PSS), and the Stress Response Inventory (SRI)^12^. Although these methods are practical and clinically useful, they are subjective and may not be suitable for continuous or real-time stress monitoring.

The human nervous system is highly complex and consists primarily of the central and peripheral nervous systems. The autonomic nervous system, which is part of the peripheral nervous system, comprises the sympathetic and parasympathetic divisions, whose balanced activity is essential for normal physiological regulation^13^. Stress disrupts this balance and can contribute to various physical and psychological health problems^14^. For this reason, physiological and neurophysiological biomarkers have become increasingly important for objective stress assessment. Signals such as electrocardiography (ECG), electrodermal activity (EDA), and electroencephalography (EEG) have been explored for this purpose^15,16^. Among these, EEG is particularly attractive because it is a noninvasive technique that directly records brain activity through electrodes placed at specific scalp locations. EEG signals reflect neural dynamics across different brain regions, including frontal, temporal, central, and occipital areas, and are commonly analyzed in terms of frequency bands such as alpha, beta, gamma, delta, and theta^17^. With the increasing availability of wearable EEG devices, such as the Muse headband, EEG-based stress monitoring is becoming more feasible in practical settings^18^.

Many authors have investigated stress detection using machine learning algorithms. In Ref. 19, stress was detected using heart rate variability (HRV) and machine learning classifiers, including KNN, MLP, and RF. Agrawal et al.^20^ conducted a comparative analysis for early stress detection using EEG signals with Random Forest and artificial neural networks. More recently, deep learning approaches such as CNNs and LSTMs have also been employed for EEG-based stress detection, where EEG frequency bands are transformed into image-like representations for classification^21^. Another study reported stress-classification accuracy of 96% using machine learning techniques^22^. More detailed discussion of these studies is provided in Sect. "Related work".

Despite these promising findings, the current literature still presents several limitations. Existing studies vary considerably in dataset size, number of EEG channels, feature extraction strategies, segmentation procedures, and validation protocols, which makes it difficult to identify which methodological choices are most effective and practical. In addition, many studies rely on either high-density EEG systems or computationally intensive deep learning models, which may limit their suitability for compact, wearable stress-monitoring applications. Furthermore, although time-domain features and segmentation techniques have been used in prior work, their combined role under a reduced-channel EEG configuration has not been systematically investigated on a relatively large public dataset. In particular, the comparative effect of overlapping and non-overlapping segmentation on classification performance remains insufficiently explored in such a setting.

To address this gap, this study investigates EEG-based stress classification using a practical and computationally efficient framework. A publicly available EEG dataset is employed, and time-domain features are extracted from four selected electrodes. Information-gain-based feature selection is then applied, and five machine learning classifiers are evaluated using both overlapping and non-overlapping segmentation strategies. Thus, the main contribution of this work lies in the systematic evaluation of a reduced-channel, time-domain, segmentation-based EEG stress-classification framework, with emphasis on practical applicability and comparative methodological analysis rather than on proposing an entirely new learning architecture.

**Fig. 1:**
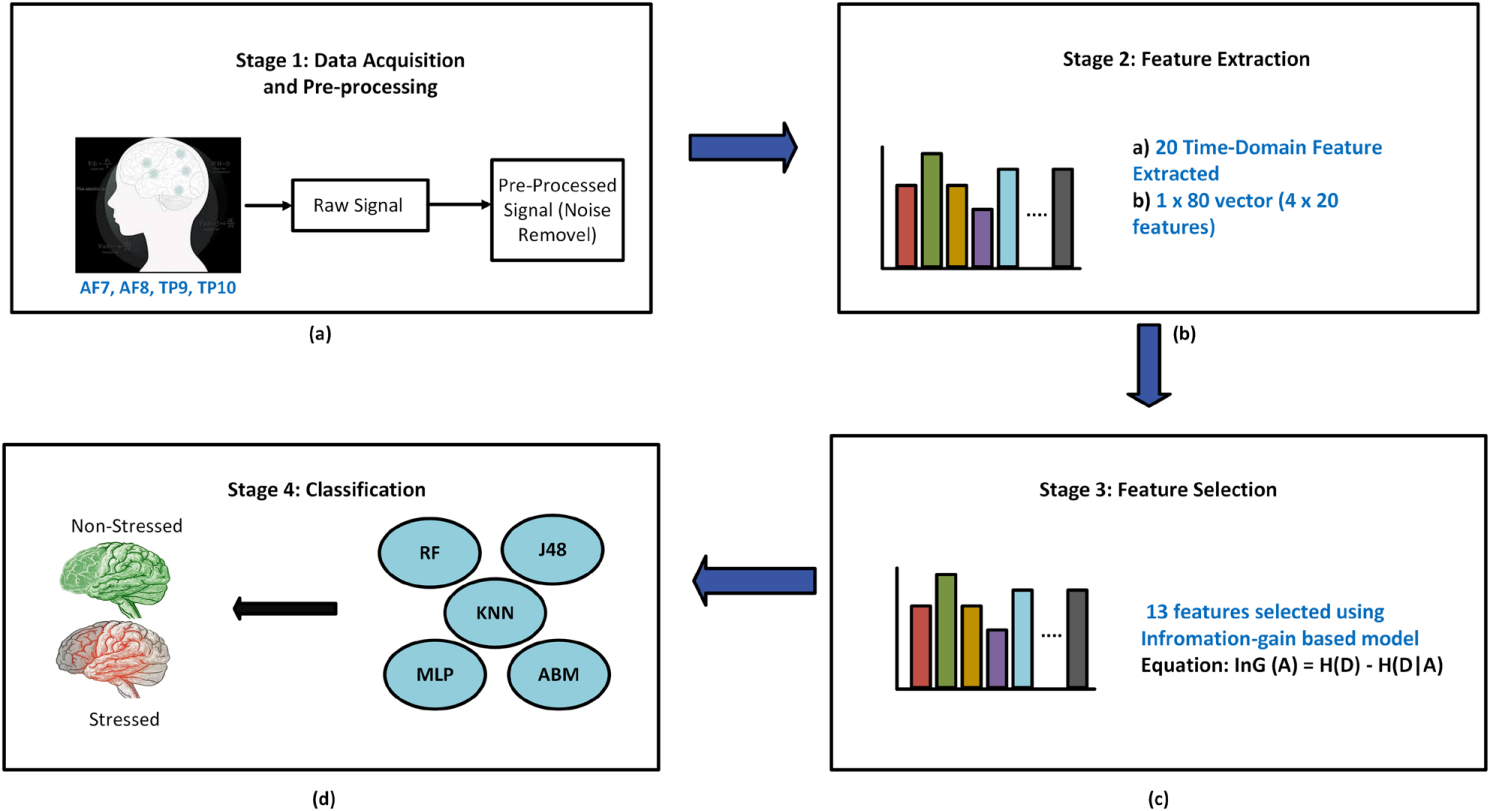
Overview of the proposed EEG-based stress detection framework using time-domain features and segmentation techniques. (**a**) Pre-processing raw EEG signals. (**b**) Feature extraction from 20 time-domain features computed using processed EEG signals. (**c**) The information gain method is used to select the most relevant features. (**d**) Five distinct machine learning classifiers are used to classify individuals into stressed vs. non-stressed.

## Related work

Stress has traditionally been assessed using clinical interviews, self-report questionnaires, and psychometric scales^23,24^. Among the most widely used instruments are the Perceived Stress Questionnaire (PSQ) and the Perceived Stress Scale (PSS), both of which have been extensively used to quantify perceived stress in behavioral and clinical studies^25^. These tools remain valuable because they are simple, inexpensive, and easy to administer. However, they are inherently subjective and depend on self-perception, recall, and reporting behavior. As a result, they are less suitable for objective and continuous monitoring, particularly in real-time or wearable health applications^26^. This limitation has motivated increasing interest in physiological and neurophysiological markers of stress, including electrocardiography (ECG), electrodermal activity (EDA), photoplethysmography (PPG), and electroencephalography (EEG)^27,28^.

Among these modalities, EEG has attracted particular attention because stress directly influences cortical activity and can therefore be studied through changes in brain dynamics. EEG is non-invasive, relatively portable, and capable of capturing stress-related neural responses with high temporal resolution^28^. Recent reviews confirm that EEG has emerged as one of the most promising modalities for stress quantification, especially when paired with machine learning and deep learning pipelines^27,29^. At the same time, these reviews emphasize major challenges in the field, including variability in experimental protocols, differences in stress-induction settings, inconsistent channel configurations, and limited reproducibility across studies^28^.

Early EEG-based stress studies largely relied on conventional machine learning models trained on handcrafted features. Asif et al. used EEG responses to music stimuli for two-level and three-level stress classification, demonstrating that machine learning could distinguish stress states from EEG recordings^18^. AlShorman et al. analyzed frontal EEG in real time and applied conventional classifiers such as SVM and Naive Bayes for mental stress detection^30^. Nirabi et al. reported stress-level detection results using KNN, SVM, Naive Bayes, and LDA, showing that classical machine learning methods can achieve competitive performance even with relatively simple pipelines^31^. Similar trends were observed by Rajendran et al., who analyzed EEG biomarkers of examination stress, and by Wen and Mohd Aris, who proposed a hybrid clustering classification framework for EEG stress analysis^32,33^. More recently, cross-context evaluation studies have shown that standard machine learning models remain attractive because of their interpretability and efficiency, although their robustness may decline when stress is assessed under heterogeneous tasks and settings^34^.

A substantial branch of the literature has focused specifically on feature engineering. In these studies, stress classification performance depends not only on the choice of classifier, but also on how EEG information is represented. Many authors have extracted statistical, temporal, spectral, or wavelet-based descriptors from EEG signals. Saeed et al. used psychological labeling and EEG-based handcrafted features for long-term stress classification, highlighting the effectiveness of feature-based learning in stress analysis^15^. Rauf and Saeed later investigated perceived stress classification using time-domain EEG features and showed that carefully selected handcrafted descriptors can yield strong results even without deep architectures^35^. Rajendran et al. used EEG as a biomarker for student stress, while Bakare et al. analyzed alpha, beta, theta, and gamma band information for mental stress detection^36,37^. Premchand et al. further showed that even wearable EEG systems with a limited number of electrodes can provide useful predictive information for stress monitoring, although classification performance may depend strongly on task context and channel placement^38^. Collectively, these studies indicate that feature engineering remains highly relevant, especially when computational efficiency, interpretability, and deployability are important design goals.

At the same time, the feature-engineering literature also reveals several unresolved issues. First, many studies use different feature sets, electrode selections, and stress-labeling strategies, making direct comparison difficult. Second, some studies report promising results on relatively small datasets or under tightly controlled laboratory conditions, which may limit generalizability^28^. Third, although segmentation is a fundamental step in EEG analysis, many studies do not explicitly compare how overlapping and non-overlapping windowing strategies affect classification results. Recent reviews have specifically highlighted the need for more systematic comparisons of preprocessing, feature extraction, and evaluation pipelines in EEG-based stress research^28,29^.

In addition to stress-specific EEG studies, several earlier EEG classification works are also relevant because they employed methodological elements similar to the present study, particularly handcrafted feature extraction, signal segmentation, and conventional machine learning-based discrimination. For example, the study “Classification of 2D and 3D videos based on EEG waves”^39^ demonstrated that EEG-derived signal characteristics can be effectively used for classification tasks involving visual perception states. Similarly, Detection of 2D and 3D Video Transitions Based on EEG Power^40^ showed that EEG power-based representations can successfully capture changes in stimulus conditions, further highlighting the usefulness of feature-based EEG analysis for pattern discrimination. Another related work, Classification of human vision discrepancy during watching 2D and 3D movies based on EEG signals^41^, also used EEG signals for the classification of perceptual differences during multimedia viewing. Although these studies do not focus on stress detection directly, they are methodologically relevant because they support the broader use of EEG-based handcrafted representations and classical classification pipelines for discriminating cognitive and perceptual states. Their inclusion helps position the present study within an existing EEG signal-classification framework and clarifies that the current work extends such established signal-processing and classification strategies toward the specific problem of stress assessment using reduced-channel EEG, time-domain features, and comparative segmentation analysis.

More recent work has increasingly adopted deep learning for stress classification. Deep models such as CNNs, LSTMs, and hybrid CNN–LSTM frameworks aim to learn stress-relevant representations directly from EEG signals or from transformed inputs such as spectrograms. Mane and Shinde proposed a hybrid LSTM–CNN model for EEG-based stress detection and reported strong performance, demonstrating the potential of temporal–spatial deep architectures^42^. Hafeez and Shakil presented a deep learning framework for EEG-based stress identification and classification, further supporting the use of data-driven representation learning^21^. More recently, Khan et al. introduced a transfer-learning-based framework using EEG spectrograms, showing that image-based representations of EEG combined with pretrained networks can further improve perceived stress classification^43^. In parallel, broader review studies have shown that deep learning is becoming increasingly dominant in EEG stress analysis because of its strong predictive capability and ability to model complex nonlinear patterns.

Despite these advances, deep learning does not fully resolve the practical limitations of EEG-based stress monitoring. Deep models often require more computational resources, larger training sets, and more complex preprocessing pipelines than conventional machine learning methods. In addition, they are typically less interpretable and may be harder to deploy in lightweight or wearable systems. Review studies published in 2024 and 2025 emphasize that high-performing models in the literature often entail substantial complexity in architectural design, input transformation, or multimodal fusion, which may limit their practicality for low-cost, real-world monitoring systems^29^. Therefore, there remains a strong need to examine whether simpler pipelines based on reduced EEG channels, computationally efficient handcrafted features, and conventional classifiers can still provide robust and meaningful performance.

Based on this synthesis, the main research gap can be stated more clearly. Although prior studies have demonstrated that stress can be detected from EEG using both handcrafted and deep features, the literature still lacks sufficient systematic analysis of practical low-complexity EEG stress classification. In particular, relatively fewer studies have jointly examined: (i) the use of a reduced channel configuration suitable for practical deployment, (ii) time-domain features that are computationally lightweight and interpretable, and (iii) the comparative impact of overlapping versus non-overlapping segmentation strategies under a common evaluation framework. The present study addresses this gap by investigating stress classification on a publicly available EEG dataset using four selected electrodes, handcrafted time-domain features, information-gain-based feature selection, and a comparative evaluation of multiple machine learning classifiers under both overlapping and non-overlapping segmentation schemes. In this way, the study is positioned not merely as another classification experiment, but as a practical and systematic investigation of efficient EEG-based stress classification.

## Dataset study

This study uses the publicly available Leipzig Study for Mind-Body-Emotion Interactions (LEMON) dataset. The dataset comprises data from approximately 228 healthy participants, including both young and older adults. Data collection was conducted between 2013 and 2015 and involved a two-day assessment protocol. The dataset includes neuroimaging, physiological, behavioral, and psychological evaluation modalities. The experimental protocol includes resting-state recordings with eyes open, followed by post-scan questionnaires^44^. In this study, we used the neuroimaging modality, specifically EEG recordings acquired using a 62-channel EEG system. The data also include psychological assessments based on 21 questionnaires and 6 cognitive tests designed to evaluate personality traits, emotional functioning, and related domains. For each subject, the dataset provides raw, processed, and localizer data.

This study focuses on classifying human stress using time-domain features extracted from EEG signals. The main contributions of this study are as follows:

- EEG data are preprocessed to enable effective stress analysis and classification. Time-domain analysis (TDA) is employed for feature extraction, and the temporal signals are examined using both overlapping and non-overlapping windowing techniques.
- Classification performance is evaluated using machine learning models, including Random Forest, Decision Tree, Multilayer Perceptron, K-Nearest Neighbor, and AdaBoostM1, to analyze the effectiveness of the selected features for stress classification.

The remainder of this paper is organized as follows. Section "Methodology" describes the proposed methodology, Sect. "Classification" presents the classification results, and Sect. "Discussion" provides a detailed comparison with previous studies.

## Methodology

The methodology for classifying human stress is discussed below and illustrated in Fig. 1. The main steps include data selection, preprocessing, feature extraction, feature selection, and classification.

### Dataset utilization

This study uses the publicly available LEMON dataset, described in Sect. "Dataset study". Since the dataset was not collected as part of the present work, only the aspects directly relevant to the current analysis are summarized here. From the full dataset, EEG recordings from the selected electrodes were used to classify stress. These channels were chosen because of their relevance to stress- and emotion-related brain activity. The EEG recordings were sampled at 2500 Hz, and participant labels were determined using the Perceived Stress Questionnaire (PSQ) scores provided with the dataset. Based on these scores, subjects were categorized into stressed and non-stressed groups according to the labeling strategy described in the following subsections.

### Pre-processing

Each participant’s EEG data is preprocessed to include only four related channels, yielding a recording of approximately 480 seconds (8 minutes). This process resulted in 211 participants. Only 211 participants have an EEG recording lasting 480 seconds. In this study, EEG signals are segmented using a windowing method. Windowing is a signal-processing technique that divides a signal into discrete segments to facilitate analysis. These windows can either overlap or not overlap. We have applied both methods to the data to gain deeper insights into which techniques overlap in stress classification and which do not. In this study, EEG signals are segmented into contiguous and non-contiguous segments over a fixed time interval. Each segmentation technique is described below.

#### Non-overlapping segmentation

It is a segmentation technique in which the entire signal is divided into consecutive, non-overlapping time-based segments. Each window is extracted by slicing non-overlapping data chunks, and no samples are reused. The subject signal is divided into 48 non-overlapping 10-second segments, which are then used to extract features.

#### Overlapping segmentation

It is a segmentation technique used in time series analysis and signal processing, in which the signal is divided into segments (windows), with each window starting before the previous one ends. Each EEG recording, with a total duration of 480 seconds per participant, was segmented into windows of length W seconds with a step size of 10 seconds. The first window starts at t = 0 sec, and each subsequent window begins 10 sec after the previous window, resulting in partial overlap between consecutive segments when the window length exceeds the step size. This sliding-window process continues until the end of the recording.

### Feature extraction

The processed data for each subject are used to extract features via time-domain analysis. These parameters include statistical measures such as the mean, median, skewness, kurtosis, variance, and standard deviation. By utilizing these features, we gained a better understanding of the EEG data for stress classification. The features extracted via time-domain analysis are described below.

#### Arithmetic mean (mean)

The Arithmetic Mean, also known as the average, is calculated by dividing the sum of all data points in a signal by the total number of points. The average value represents the signal’s central tendency over the specified duration. Equation (1) shows the mathematical expression of the arithmetic mean.

1 $$\begin{aligned} \text {Mean} = \frac{1}{n} \sum _{i=1}^{n} x_i, \end{aligned}$$

where Mean is the average of the samples, *n* represents the total number of samples in the entire signal, and $x_i$ is the individual sample in the whole signal.

#### Standard deviation (std)

A crucial statistical measure that quantifies the dispersion of signal values around their mean value. Lower values of standard deviation indicate that signal values are clustered around the Mean, and higher values of standard deviation indicate that signal values are far away from the mean value. Mathematically, standard deviation is described by Eq. (2).

2 $$\begin{aligned} \text {Std} = \sqrt{\frac{1}{n-1} \sum _{i=1}^{n} \left( x_i - \bar{x} \right) ^2}, \end{aligned}$$

where Std represents standard deviation, $x_i$ is the single point in the signal, *n* is the total number of samples, and $\bar{x}$ is the mean value of the entire signal values.

#### Kurtosis (Kurt)

In signal processing, Kurtosis is a measure of how peaked a signal’s probability distribution is relative to a normal distribution. It is used for quantifying the non-Gaussian behavior of signals. Equation (3) shows the mathematical expression for kurtosis, which is called Pearson’s coefficient^35^.

3 $$\begin{aligned} \text {Kurt} = \frac{1}{n} \sum _{i=1}^{n} \left( \frac{x_i - \bar{x}}{\text {Std}} \right) ^{4}, \end{aligned}$$

where Kurt is the Kurtosis measure, $x_i$ is the sample of the signal, $\bar{x}$ is the mean value, $\text {Std}$ is the standard deviation, and *n* represents the total number of samples in the entire signal.

#### Skew (skew)

Skew is a measure of the difference in signal arrival times, particularly across components within the system. The value of skew can be either positive, negative, or zero. If skew > 0, it indicates a right skew, meaning the tail of the signal is on the right side. If skew < 0, it indicates a left skew, where the tail of the signal is on the left side. If skew is nearly zero, it indicates a symmetrical distribution of the signal in the system. Many formulas calculate skew, but the formula we have used is shown in Eq. (4), which is called Pearson’s coefficient.

4 $$\begin{aligned} \text {Skew} = \frac{1}{n} \sum _{i=1}^{n} \left( \frac{x_i - \bar{x}}{\text {Std}} \right) ^{3}, \end{aligned}$$

where Skew is the statistical measure, *n* is the total number of samples, $x_i$ is a single sample in the entire signal, $\bar{x}$ is the mean value, and $\text {Std}$ is the standard deviation.

#### Peak to peak signal value (PP)

The peak-to-peak signal value is the difference between the signal’s maximum and minimum, measuring the vertical distance between the highest and lowest points. It is also called the PP value. The peak-to-peak value for the signal is usually calculated using Eq. (5).

5 $$\begin{aligned} \text {PP} = \text {Amp}_{\text {max}} - \text {Amp}_{\text {min}}, \end{aligned}$$

where *PP* is the peak to peak signal value, $\text {Amp}_{\text {max}}$ is the maximum value of the signal, and $\text {Amp}_{\text {min}}$ is the minimum value of the signal.

#### Peak to peak time (PPT)

Peak-to-peak time is the interval between the signal’s highest and lowest troughs, a measure of its amplitude. It is represented by Eq. (6).

6 $$\begin{aligned} \text {PPT} = \left| t_{\text {max}} - t_{\text {min}} \right| , \end{aligned}$$

where PPT is peak to peak time, $t_{\text {max}}$ is the time for maximum amplitude, $t_{\text {min}}$ is the time for minimum amplitude.

#### Peak to peak slope (PPS)

Peak to peak slope refers to the line or slope connecting the highest and lowest troughs of the signal. It quantifies the signal’s slope. This time domain feature is represented by the following Eq. (7).

7 $$\begin{aligned} \text {PPS} = {\frac{\textrm{PP}}{\textrm{PPT}}}, \end{aligned}$$

where PPS is the peak to peak slope of the signal, PP is the peak to peak value of the signal, and PPT is the peak-to-peak time of the signal.

#### Minimum latency ($L_{\text {min}}$)

The minimum time delay of the input signal is called the minimum latency. To determine the signal’s minimum latency, Eq. (8) is used.

8 $$\begin{aligned} L_{\text {min}} = t \big |_{X = X_{\text {min}}}, \end{aligned}$$

where $L_{\text {min}}$ is the minimum latency (time at which the signal reaches its minimum), $X_{\text {min}}$ is the minimum value of the signal, and *t* is the corresponding time index.

#### Maximum amplitude ($\text {Amp}_{\text {max}}$)

The value of the signal at which it reaches its maximum value is called the Maximum Amplitude. This is the point at which the signal has its largest value. Equation (9) is used to calculate maximum amplitude.

9 $$\begin{aligned} \text {Amp}_{\text {max}} = \max (X), \end{aligned}$$

where $\text {Amp}_{\text {max}}$ is the maximum amplitude of the signal and *X* is the EEG signal.

#### Minimum amplitude ($\textrm{Amp}_{\min }$)

The value at which the signal reaches its minimum value is referred to as the minimum amplitude. It is the point at which it has its smallest value. Equation (10) is used to calculate the minimum amplitude of the signal.

10 $$\begin{aligned} \textrm{Amp}_{\min } = \min (X). \end{aligned}$$

#### Absolute latency to amplitude ratio (ALAR)

The ALAR is a time-domain feature used as an indicator of neurological function in EEG, biomedical, and EMG studies. It measures how quickly a signal reaches its peak amplitude, relative to the peak’s magnitude. ALAR is calculated using Eq. (11).

11 $$\begin{aligned} \textrm{ALAR} = \frac{|X_{\max }|}{L_{\max }}, \end{aligned}$$

where $L_{\max }$ is the maximum latency (time at which the signal reaches its maximum), and $X_{\max }$ is the maximum amplitude of the signal.

#### Mean of absolute values of first difference (MDIF1)

Mean of absolute values of first difference is the computed average of the magnitude of change between consecutive samples of a signal, irrespective of whether the change is positive or negative. If the MDIF1 value is large, it changes sharply. If the MDIF1 value is small, the change occurs gradually. MDIF1 is expressed by Eq. (12).

12 $$\begin{aligned} \text {MDIF1} = \frac{1}{N} \sum _{n=1}^{T} \left| Sa(n) - Sa(n-1) \right| , \end{aligned}$$

where MDIF1 is the Mean of the absolute values of the first difference. $N$ is the total number of samples. $T$ is the total number of points in the time series. $Sa(n)$ is the value of the signal at $n$. $Sa(n) - Sa(n-1)$ is the difference between consecutive points.

#### Mean of absolute values of second difference (MDIF2)

It is a time-domain feature that calculates the average magnitude of the differences between consecutive points in a time series. It is the acceleration or the rate of change of velocity. MDIF2 is calculated by using Eq. (13).

13 $$\begin{aligned} \mu _{\nabla \nabla } = \frac{1}{N} \sum _{n=2}^{T-1} \left| Sa(n+1) - 2\,Sa(n) + Sa(n-1) \right| , \end{aligned}$$

where: $\mu _{\nabla \nabla }$ is the Mean of the absolute values of the second difference. *N* is the total number of samples in the signal. *T* is the total number of points. $Sa(n+1) - 2Sa(n) + Sa(n-1)$ is the second difference between consecutive samples.

#### Maximum latency ($L_{\max }$)

The signal’s maximum latency is the time at which it reaches its maximum amplitude. To calculate the maximum latency, Eq. (14) is used.

14 $$\begin{aligned} L_{\text {max}} = t \big |_{X = X_{\text {max}}}, \end{aligned}$$

where $L_{\text {max}}$ is the maximum latency (time at which the signal reaches its maximum), $X_{\text {max}}$ is the maximum amplitude of the signal, and *t* is the corresponding time index.

#### Normalized mean of the absolute value of first difference (Mdif1norm)

It is a domain feature used to calculate the normalized average magnitude of change between consecutive points. Normalization facilitates comparison of change magnitudes across scales. Equation (15) is used to calculate Mdif1norm.

15 $$\begin{aligned} \text {Mdif1norm} = \frac{1}{N-1} \sum _{n=2}^{T} \left| x_n - x_{n-1} \right| , \end{aligned}$$

where *Mdif*1*norm* is the normalized Mean of the absolute value of the first difference, N is the total number of samples, $x_n$ is the individual point in the sample, and T is the total number of points in the time series.

#### Normalized mean of the absolute value of second difference (Mdif2norm)

It is a time-domain feature used to compute the average magnitude of differences or acceleration, scaled by a normalization factor. Mdif2norm is calculated by using Eq. (16).

16 $$\begin{aligned} \textrm{Mdif2norm} = \frac{1}{N-2} \sum _{n=2}^{T-1} \left| x_{n+1} - 2x_n + x_{n-1} \right| , \end{aligned}$$

where $\textrm{Mdif2norm}$ is the normalized Mean of the absolute value of the second difference, *N* is the total number of samples, *T* is the total number of points in the time series, and $x_n$ is the signal value in the index *n*.

#### Energy (E)

Energy is a time-domain measure of the total strength of a signal over its duration. It represents the accumulated power of the signal. Equation (17) is used to measure the energy of the signal.

17 $$\begin{aligned} E = \sum _{n=1}^{T} |x[n]|^2. \end{aligned}$$

Here, E represents the energy of the signal, T is the total number of points in the time series, and x[n] is the individual point of the signal.

#### Normalized energy ($E_{\text {norm}}$)

Normalized energy is the scaled version of energy, enabling comparison of signals of different sizes. We can calculate the normalized energy using Eq. (18).

18 $$\begin{aligned} E_{\text {norm}} = \frac{1}{T} \sum _{n=1}^{T} |x[n]|^2, \end{aligned}$$

where $E_{\text {norm}}$ is the normalized energy of the signal, *T* is the total number of points, and *x*[*n*] is the individual point of the signal.

#### Latency to amplitude ratio (LAR)

The latency-to-amplitude ratio is a time-domain feature that compares the signal’s latency to its total strength. It is calculated by Eq. (19).

19 $$\begin{aligned} \textrm{LAR} = \frac{L}{\textrm{Amp}}, \end{aligned}$$

where LAR is the latency to amplitude ratio, L is the latency of the signal, and Amp is the amplitude of the signal.

#### Entropy (Ent)

The entropy of a signal is a statistical measure of its randomness. Low entropy means that the signal is structured and predictable. High entropy indicates that the signal is more distorted and random. The entropy is measured by Eq. (20).

20 $$\begin{aligned} \textrm{Ent} = \sum _{n=1}^{N} c_n \log (c_n), \end{aligned}$$

where $\textrm{Ent}$ is the entropy of the signal, *N* is the total number of samples of the signal, and $c_n$ is the probability of the *nth* bin, estimated as the fraction of samples in that bin over the total number of samples.

### Feature selection

The information gain-based model is an entropy-based metric that is used for feature selection. It quantifies the extent to which knowledge of the features reduces uncertainty about the target class. It selects only those high-ranking features that are helpful and distinguish between stressed and non-stressed words. Entropy is measured and explained by Eq. (21).

21 $$\begin{aligned} H(Y) = - \sum _{i=1}^C p(y_i) \log _2 p(y_i), \end{aligned}$$

where *H*(*Y*) is the entropy of the target variable, *C* is the number of categories in the dataset, and $p(y_i)$ is the probability of the *i*th element. Conditional entropy quantifies the remaining uncertainty in a dataset after splitting it on a specific feature. It is calculated by Eq. (22).

22 $$\begin{aligned} H(D \mid A) = \sum _{v \in A} \frac{|D_v|}{|D|} H(D_v), \end{aligned}$$

where $H(D \mid A)$ is the entropy of the dataset *D* after splitting it into the feature *A*, |*D*| is the total number of data points, and $|D_v|$ is the number of data points that have value *v* for the feature *A*. Finally, the information gain is calculated using Eq. (23).

23 $$\begin{aligned} \textrm{InG}(A) = H(D) - H(D \mid A), \end{aligned}$$

where $\textrm{InG}(A)$ is the approach based on the gain of information for the specific feature *A*, *H*(*D*) is the entropy of dataset *D*, and $H(D \mid A)$ is the entropy of dataset *D* after splitting it into features *A*.

**Fig. 2:**
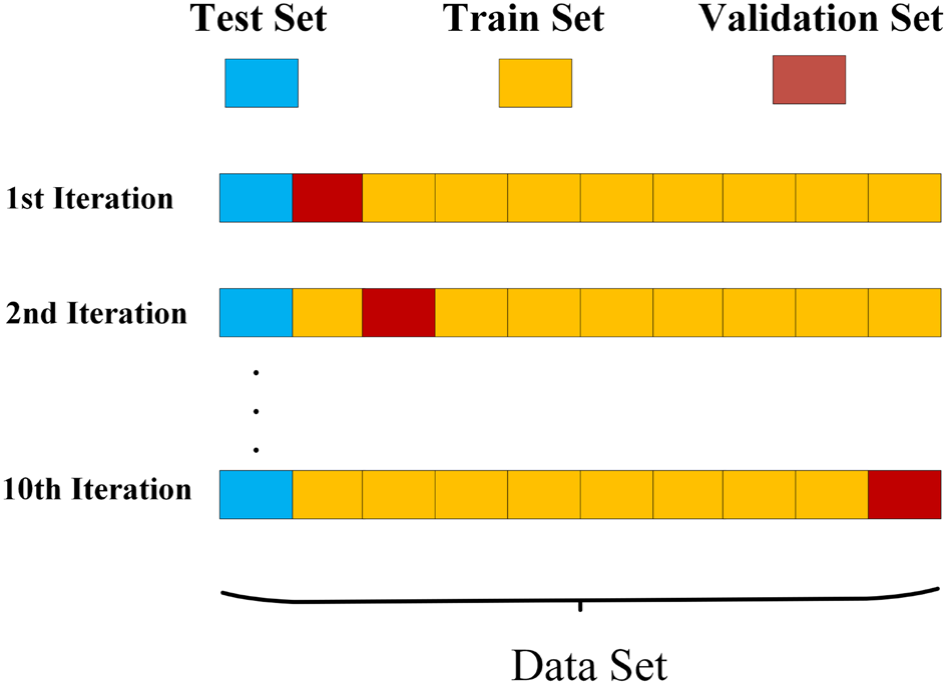
Illustration of the ten-fold cross-validation process used for model evaluation.

## Classification

The data, after all preprocessing, is used to classify stress into two classes: stressed or non-stressed. A 10-fold cross-validation technique is employed for the classification task with machine learning classifiers. This technique is the most widely used in machine learning to assess a classifier’s performance and its ability to produce accurate results on unseen data. The model is first trained on the training data and then evaluated on unseen data. Using this technique, the dataset is divided into ten equal subsets. Out of these ten folds, nine are used for training of data and one fold is used for testing of data (see Fig. 2). The performance metrics used to assess the classifier’s results are precision, accuracy, recall, and F-measure. These metrics, computed after each iteration, represent the average across the overall results. We have used five distinct classifiers for the classification task. Details for each classifier’s specification are given in the following section.

**Fig. 3:**
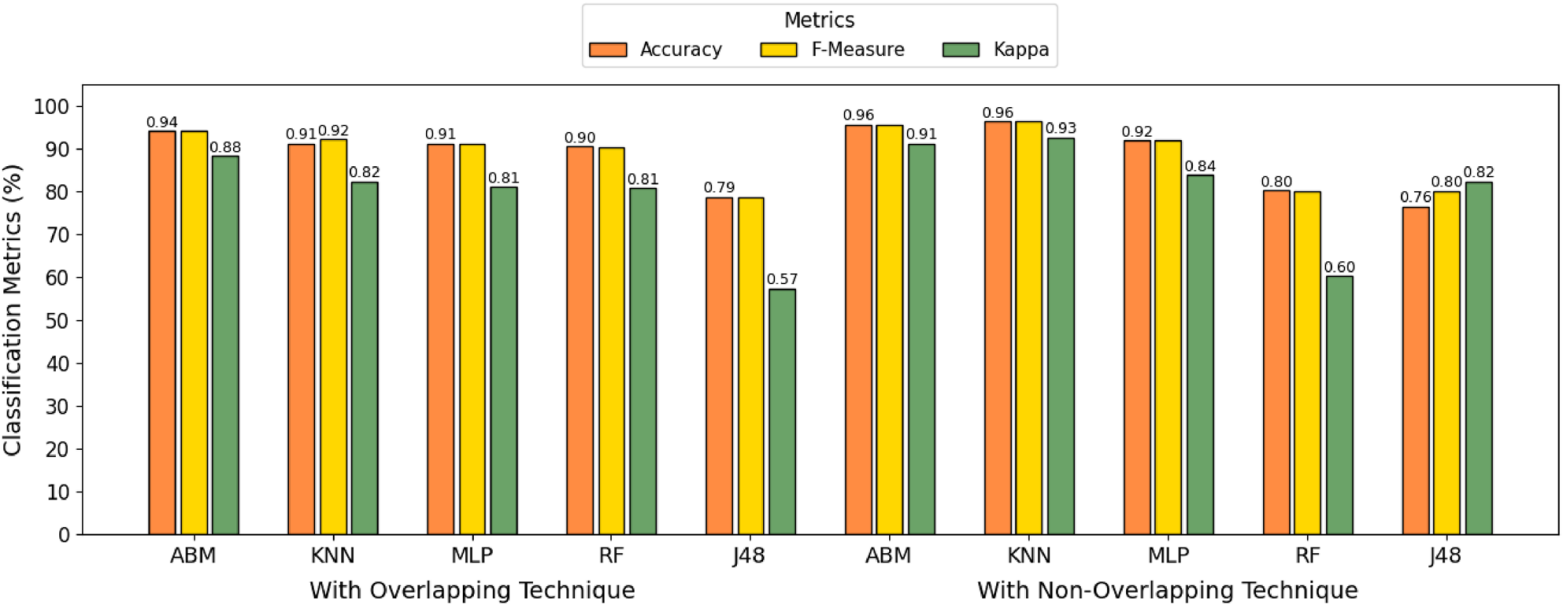
Accuracy trends for the five classifiers used for stress detection using EEG dataset.

**Table 1:** Hyperparameter settings for non-overlapping and overlapping segmentation techniques.

Classifier	Non-overlapping technique	Overlapping technique
Random Forest (RF)	Iterations=100, MaxDepth=10, Seed=1	Iterations=100, Max Depth=10, Seed=1
J48(C4.5)	C=0.15, Folds=3, MinNumObj=5	C=0.15, Folds=5, MinNumObj=5
KNN	K=5, Weight=1/Distance, nearestsearchalgorithm = Linear Search	K=5, Weight=1/Distance, nearestsearchalgorithm = Linear Search
MLP	L=0.3, M=0.2, Epochs=500, VT=20, VS=0	L=0.3, M=0.2, Batch Size=100
AdaBoostM1	Iterations=100, WeightThreshold=100, Classifier = Decision Stump	Iterations=200, WeightThreshold=200, Classifier = Decision Stump

### Random forest (RF)

A Random Forest (RF) classifier combines multiple decision trees and is an ensemble learning technique for classification. Each tree is trained independently on each data subset, thereby reducing the risk of overfitting and improving the model’s generalization performance. In our proposed study, RF is employed to classify perceived stress from EEG signals. Input features are extracted by applying signal-processing and feature-extraction methods to the EEG signals.RF used bagging during training. Each tree is trained on a bootstrapped copy of the training set and on a random subset of features.

After all the trees have been trained, a fresh EEG recording is labeled using majority voting. This technique ensures stable performance in the presence of noise and high-dimensional feature spaces. Mathematically, RF is expressed by the following Eq. (24).

24 $$\begin{aligned} \textrm{RF}\left( x\right) =\frac{1}{M}\sum _{i=1}^{M}{T_i\left( x\right) }, \end{aligned}$$

where *RF*(*x*) is the Random Forest for input *x*, *M* is the total number of decision trees, $T_i(x)$ is the output of the decision tree for the input *x*.

### Decision tree (DT)

The Decision Tree (DT) method is widely used for classification in supervised learning tasks. It is simple, interpretable, and can easily handle both numerical and categorical data. Its working principle is based on splitting the data into subsets using the most informative feature at each node of the tree. The process continues until leaf nodes are formed, which represent the label class. Pruning strategies are applied to mitigate the risk of overfitting during classification. Our proposed study used the J48 classifier, an extension of the DT framework that handles missing and null values and employs advanced pruning techniques to improve generalization. Mathematically, a Decision Tree is represented by Eq. (25).

25 $$\begin{aligned} h\left( x\right) =\sum _{j=1}^{N}a_j\cdot I\left( x\in B_j\right) , \end{aligned}$$

where *N* is the total number of leaf nodes, $I\left( x\in B_j\right)$ is the indicator function, and $a_j$ is the predicted output.

### Multilayer perceptron (MLP)

The Multilayer Perceptron (MLP) is a supervised learning technique that utilizes an input layer, one or more hidden layers, and an output layer. Each layer consists of connected neurons (processing units). Each neuron computes the weighted sum of its inputs and passes the result to the output layer via nonlinearar activation function. MLPs are well-suited to EEG-based classification tasks because they learn subtle patterns embedded in brain signals. Mathematically, MLP is calculated by Eq. (26).

26 $$\begin{aligned} z_j^{\left( l\right) }=\sum _{i=1}^{h^{\left( l-1\right) }}{W_{ji}^{\left( l\right) }a_i^{\left( l-1\right) }}+b_j^{\left( l\right) }, \end{aligned}$$

where $z_j^{\left( l\right) }$is the weighted sum at neuron j at the layer *l*, $W_{ji}^{\left( l\right) }$ is the associated weight, $a_i^{\left( l-1\right) }$ is the activation function of neuron *i* at the previous layer $l-1$, $b_j^{\left( l\right) }$ is the bias unit of neuron *j* at layer *l*.

### K-nearest neighbors (KNN)

K-nearest neighbors is a distribution-free algorithm that performs regression and classification. It determines the output by examining the k-nearest examples in the training data. Nearest points are often measured using Euclidean distance. In classification, the output label is determined by majority voting. It is a lazy learning algorithm; it doesn’t require any explicit training because it can predict output values using stored data. Euclidean formula is used to calculate the distance between the new point $x_n$ and the existing sample $x_i$, which is shown by the Eq. (27).

27 $$\begin{aligned} d\left( x_\textrm{n},x_i\right) =\sqrt{\sum _{j=1}^{n}\left( x_{\textrm{n},j}-x_{i,j}\right) ^2}, \end{aligned}$$

where *n* is the total number of samples, $x_n$ and $x_i$ are the new and existing points for the *jth* feature value, and $d\left( x_n,x_i\right)$ is the distance between the new and existing points.

### AdaBoostM1 (ABM)

Adaptive Boosting (AdaBoostM1) is an ensemble learning algorithm that aggregates multiple weak classifiers into a single strong classifier. A weak learner outperforms random guessing, but when combined via boosting, the resulting accuracy increases. This algorithm operates iteratively. In the first iteration, a weak classifier is trained on the dataset’s weighted samples. Based on these results, the weights of misclassified samples are adjusted to give more attention to these cases. This process repeats until the specified number of iterations or the desired accuracy is achieved. AdaBoost is calculated by Eq. (28).

28 $$\begin{aligned} H\left( x\right) =\textrm{sign}\left( \sum _{t=1}^{T}\alpha _t\cdot h_t\left( x\right) \right) , \end{aligned}$$

where $H\left( x\right)$ is the final prediction for input *x*, *T* is the total number of weak classifiers combined, $\alpha _t$ is the weight assigned to the prediction, $h_t\left( x\right)$ is the prediction of the classifier.

**Table 2:** Selected features for non-overlapping segmentation technique using information-gain based method.

Electrodes	Features selected
AF7	Lmin, MDIF2
AF8	Lmin
TP7	MDIF1, Energy, Normalized energy
TP8	Lmin, Mdif2norm, Entropy

**Table 3:** Selected features for overlapping segmentation technique using information-gain based method.

Electrodes	Features selected
AF7	MDIF2
AF8	Mean, PPS, ALAR, Lmax, Entropy, LAR
TP7	Mean
TP8	Mean

**Table 4:** Performance metrics of the five classifiers used for stress detection.

Classifier	Technique	Accuracy	F-measure	Kappa	Recall	Precision	ROC
ABM	Overlapping	94.12%	0.94	0.88	0.94	0.94	0.96
	Non-Overlapping	95.59%	0.96	0.91	0.96	0.96	0.98
KNN	Overlapping	91.18%	0.91	0.82	0.91	0.91	0.97
	Non-Overlapping	96.32%	0.96	0.93	0.96	0.96	0.99
MLP	Overlapping	91.18%	0.91	0.81	0.91	0.91	0.96
	Non-Overlapping	91.91%	0.92	0.84	0.92	0.92	0.94
RF	Overlapping	90.44%	0.90	0.81	0.90	0.91	0.98
	Non-Overlapping	80.15%	0.80	0.60	0.80	0.80	0.90
J48	Overlapping	78.68%	0.79	0.57	0.79	0.79	0.82
	Non-Overlapping	76.47%	0.80	0.82	0.77	0.77	0.79

Where ROC is the receiver operating characteristic.

## Results

### Data labeling

Each subject was classified as stressed or non-stressed based on PSQ scores. Following prior perceived-stress classification studies, threshold values were defined using the mean and standard deviation of PSQ scores, as given in Eq. (29).

29 $$\begin{aligned} TP = \mu \pm \frac{\sigma }{2}. \end{aligned}$$

From the original 228 participants, 211 subjects were retained after preprocessing. Using this criterion, the mean PSQ score was 30.20 and the standard deviation was 15.52, resulting in thresholds of 22.44 and 37.96. Subjects with PSQ scores below $\mu - \frac{\sigma }{2}$ were labeled as non-stressed, whereas subjects with PSQ scores above $\mu + \frac{\sigma }{2}$ were labeled as stressed. Accordingly, 75 subjects were labeled as non-stressed, and 61 subjects were labeled as stressed, while subjects with intermediate PSQ scores between these thresholds were excluded from the binary classification setting.

This labeling strategy was adopted to create a clearer binary discrimination problem by reducing ambiguity in subjects whose stress scores fall near the central range, consistent with prior perceived-stress classification studies^35^. However, this choice also reduces the effective sample size and may increase class separability, which can lead to optimistic classification performance. Therefore, the present formulation should be interpreted as a constrained binary stress-classification setting rather than a complete modeling of the full stress continuum. Future work should investigate alternative thresholding schemes, as well as ordinal or multi-level stress-labeling strategies that retain intermediate cases.

### Feature selection

We have applied an information gain-based model to select the top-ranked features for classification Table 1. Using the information gain method, 10 features were selected from the feature vector. Selected features for the non-overlapping segmentation technique are listed in Table 2. For the overlapping segmentation technique, selected features are shown in Table 3.

**Fig. 4:**
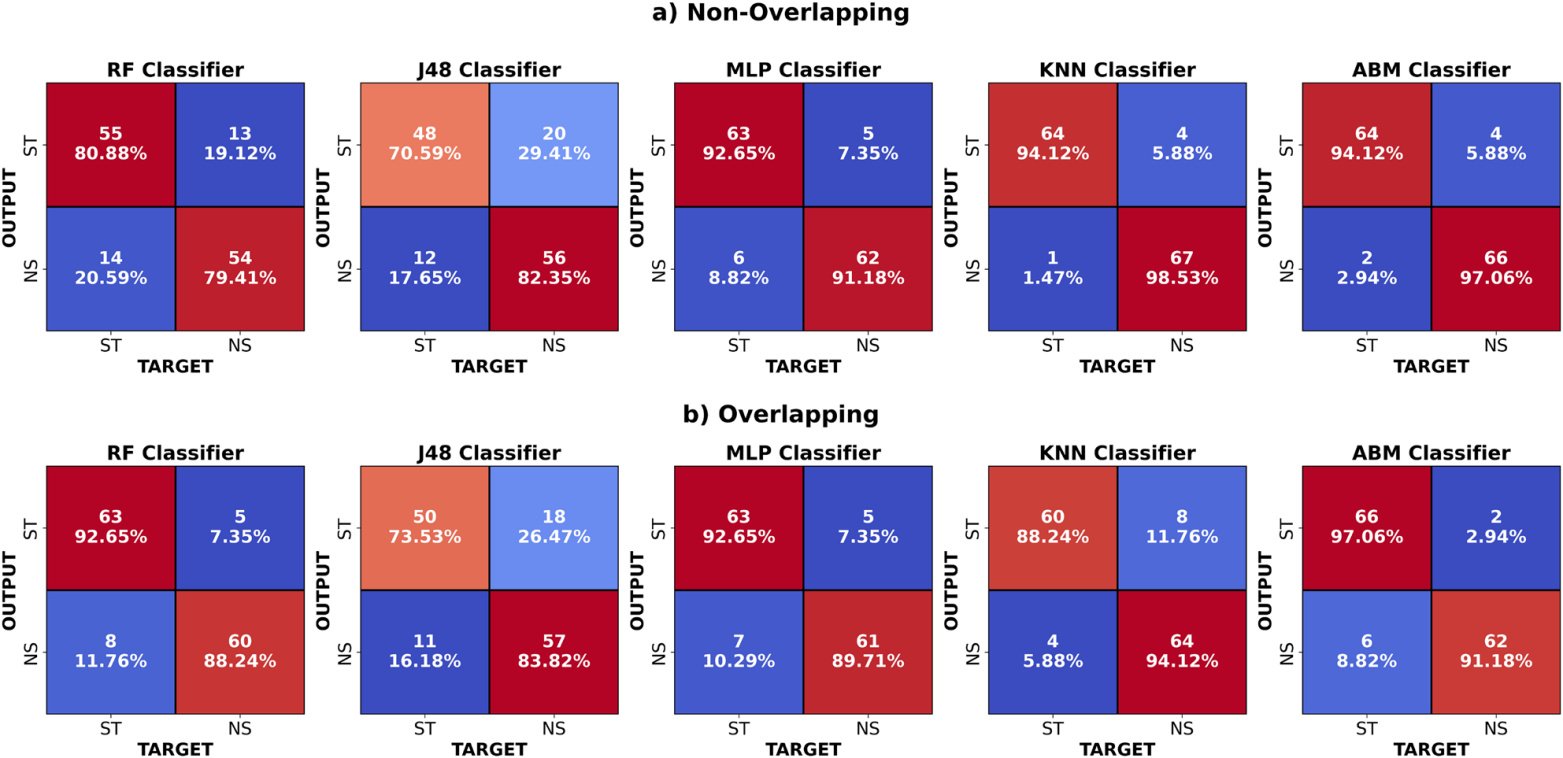
Confusion matrices for the proposed EEG-based framework for stress classification under two segmentation techniques. (**a**) Non-overlapping technique: Each classifier is evaluated using non-overlapping EEG segments, with correctly classified samples labeled as stressed (ST) or non-stressed (NS). (**b**) Overlapping technique: The classifiers are evaluated on overlapping EEG segments, demonstrating how increased sample density affects the distribution of samples classified as stressed (ST) and non-stressed (NS).

### Classification performance

In this study, we classified participants as stressed or non-stressed using the feature-selection methodology described in Sect. "Feature selection". Classifier performance has been evaluated using 10-fold cross-validation. Accuracy, F-measure, Precision, and Recall are the metrics by which our model’s performance has been assessed in Fig. 3. In addition to conventional performance metrics, Golden Distance was computed as a complementary unified measure for classifier comparison. The preprocessed data were used to extract 20 temporal-domain characteristics from the four electrodes. Classifiers are used for the classification of stress as described in Sect. "Classification". The performance of each classifier for the non-overlapping and overlapping techniques is shown in Table 4.

In the non-overlapping technique, the KNN algorithm achieves an accuracy of 96.32%, as shown in Table 4. This algorithm has achieved the highest accuracy and the best classification results. An accuracy of 95.59% is reported by the ABM classifier using 10-fold cross-validation. According to Table 4, RF, J48, and MLP achieved accuracies of 80.14%, 76.47%, and 91.91%, respectively.

In the overlapping method, the highest accuracy, i.e. 94.12%, is recorded by the ABM classifier by using 10-fold cross-validation, as shown in Fig. 2. The RF, J48, MLP, and KNN classifiers achieved classification accuracies of 90.44%, 78.68%, 91.18%, and 91.18%, respectively.

Figure 4 shows the visual results of all classifiers in the form of a confusion matrix. The confusion matrix is used to evaluate a model’s classification performance by comparing actual and predicted instances. Table presents all classified and misclassified instances across all classifiers. For example, among 136 cases, the MLP classifier using the overlapping methodology correctly classified 124 and incorrectly classified only 12. The study^35^ reported a classification accuracy of 93.8% by using a Random Forest model with the overlapping technique for perceived stress detection (Table 5).

**Table 5:** Overview of prior studies based on stress classification using EEG signals.

Ref, year	Electrodes	Duration	Classifier	Accuracy
15, 2020	4	24 min	LDA	85.60%
25, 2022	8	3 min	DT	87.00%
33, 2023	4	24 min	SVM	90.55%
36, 2023	10-20	–	XGBoost	98.00%
34, 2023	16	20 min	MLP	96.01%
35, 2023	4	–	ABM	91.50%
38, 2024	4	3 min	RF	93.70%
**Proposed, 2025**	**4**	**8 min**	**KNN**	**96.32%**

Significant values are in bold.

To provide a more comprehensive evaluation of classifier performance, Golden Distance was also considered in this study. Roza first emphasized the importance of unified evaluation measures for classification systems^45^, and Golden Distance later extended this idea by defining a single metric that reflects the overall distance of a classifier from ideal performance^46^. Therefore, Golden Distance was used here as a complementary metric alongside accuracy, precision, recall, F-measure, kappa, and ROC to support a more balanced comparison across classifiers. Golden Distance jointly considers multiple performance criteria in a single score, with lower values indicating better, more balanced classification performance. The Golden Distance results are presented in Table 6.

As shown in Table 6, the KNN classifier with the non-overlapping segmentation technique achieved the lowest Golden Distance value of 0.0784, indicating the best overall and most balanced performance among all evaluated models. AdaBoostM1 with non-overlapping segmentation ranked second with a Golden Distance of 0.0907, followed by AdaBoostM1 with overlapping segmentation. These results are consistent with the standard evaluation metrics and further support the effectiveness of the non-overlapping segmentation strategy in the proposed framework.

**Table 6:** Golden distance analysis of classifier performance.

Classifier	Technique	Av-NAV	Av-DCO	Golden distance
KNN	Non-Overlapping	0.9226	0.0125	0.0784
ABM	Non-Overlapping	0.9106	0.0155	0.0907
ABM	Overlapping	0.8714	0.0183	0.1299
MLP	Non-Overlapping	0.8276	0.0236	0.1740
KNN	Overlapping	0.8192	0.0316	0.1836
MLP	Overlapping	0.8131	0.0325	0.1897
RF	Overlapping	0.8108	0.0354	0.1925
J48	Non-Overlapping	0.6174	0.0137	0.3829
RF	Non-Overlapping	0.6124	0.0646	0.3930
J48	Overlapping	0.5728	0.0617	0.4316

A lower golden distance indicates better, more balanced classification performance.

## Discussion

In our proposed methodology, we used a 62-channel EEG system to record data from the prefrontal and temporal brain regions. The electrodes used in the study are AF7, AF8, TP7, and TP8. Using EEG data, we have classified human stress. The recorded dataset is first preprocessed and later used. The maximum recording duration of EEG signals we used was 8 minutes. This 8-minute recording is long enough to capture robust EEG patterns, but it may challenge participants’ ability to maintain a consistent state of mind. Using five distinct classifiers, we achieved an accuracy of 96.32% with the non-overlapping technique, which is notable. This achievement excels in stress classification research.

In this study, EEG signals are segmented using both overlapping and non-overlapping techniques, and model performance is evaluated using 10-fold cross-validation, as shown in Fig. 2. All segmented samples were randomly distributed into 10 folds. One fold is used for training, and the remaining nine folds are used for testing in each iteration. As a result, the same subjects appear in both the test and training data. This evaluation strategy enables efficient utilization of the available data. It is widely adopted in EEG-based stress classification studies to assess the discriminative capability of extracted features and machine-learning classifiers under controlled experimental conditions. While this approach may yield optimistic performance estimates due to potential subject-level correlations, the primary objective of this work is to analyze feature effectiveness and classifier behavior rather than cross-subject generalization.

Furthermore, dataset size, experimental duration, and methodology are crucial factors to consider for the robustness, generalizability, and reliability of results. This study demonstrates that high classification accuracy can be achieved without using extensive hardware setups. The compact design and limited usage of EEG channels make it suitable for a real-world deployment of a health monitoring system. This minimal setup reduces hardware complexity and user discomfort, both of which are crucial for sustained use in daily life. Such systems can be integrated into mobile applications or wearable devices, such as headbands, for real-time stress monitoring. Table 5 shows the results of previously proposed methods for stress classification using EEG signals. This comparison is crucial for contextualizing our findings within the existing literature and highlighting the results of our methodology. Our proposed study focuses on a more in-depth analysis of electrode selection, optimized classifier selection, and experimental duration.

Using time-domain analysis for feature extraction is a widely employed strategy for classification tasks. The most valuable features are Peak-to-Peak Signal Value, Mean, Maximum Latency, Entropy, Mean Latency-to-Amplitude Ratio, and ALAR. The AF8 electrode uses these features in the overlapping segmentation technique. Studies show that the AF8 electrode significantly contributes to stress classification and detection, as this brain region is involved in regulating stress responses^47^. In our study, we used both non-overlapping and overlapping segmentation techniques. We have found that the non-overlapping technique has produced favorable results, with a classifying accuracy of 96.32%, as shown in Table 4. When using the overlapping technique, the highest classification accuracy is 94.11%, as shown in Table 4. In our proposed study, we employed 10-fold cross-validation to evaluate the classifier. This matches the experimental study in Ref. 33. This study reveals that the prefrontal region makes a significant contribution to stress detection, achieving 98% accuracy with an SVM classifier. In conclusion, our study has developed an efficient method for classifying mental stress using a streamlined 4-channel EEG system. Our experimental system aligns well with state-of-the-art techniques^18,49,50^, and others, in EEG-based stress classification. Our proposed methodology, which utilizes a 62-channel EEG system, aligns with the studies^35,51,52,53^. This study’s accuracy, 96.3235%, notably exceeds the 75% accuracy reported for k = 7 in Ref. 54. Most studies have favored the Support Vector Classifier (SVC), K-Nearest Neighbor (KNN), and Logistic Regression (LR) for stress classification^55^.

Compared with the study^43^, which proposed a transfer-learning-based framework for perceived stress detection in which EEG signals were converted into spectrograms and fine-tuned deep learning models classified stress, this study focuses on using spectrograms for perceived stress detection. Their transfer learning method achieved 95.80% accuracy for two-level classification, whereas it was 86.02% for three-level classification. However, the above study shows that an accuracy of 96.32% can be achieved using KNN with non-overlapping segmentation. Despite a simpler feature set, our study demonstrates that lightweight machine-learning models, when combined with carefully selected EEG time-domain features, achieve stress-classification performance comparable to that of more complex deep-learning methods.

**Algorithm 1:**
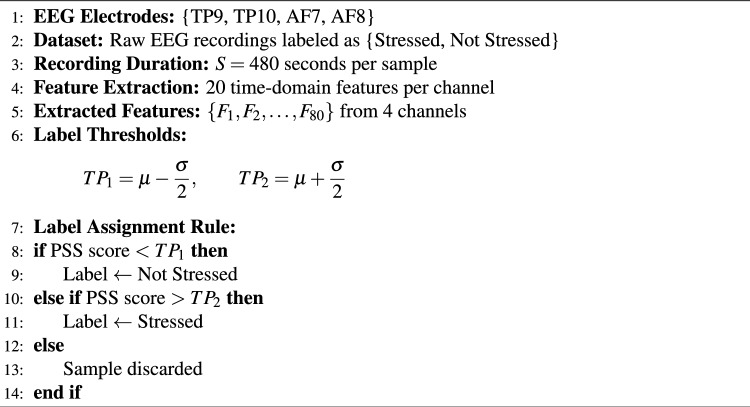
Stress classification using time domain analysis.

## Future enhancements

Our proposed study yields promising results by utilizing a minimal number of electrodes for data collection. We have extracted 20 time-domain features for evaluating model performance, but these may not capture the complex dynamics of stress-related brain activity. In the future, we can explore Multiscale Fluctuation Dispersion Entropy (MFDE) and Multivariate Multiscale Dispersion Entropy (mvMDE) to achieve superior classification performance. Moreover, integrating environmental factors and more physiological signals, such as ECG and GSR, can improve this field of research. A limitation of this study is that it lacks subject-wise or leave-one-subject-out validation, which may overestimate results due to specific subject patterns in EEG signals. In this way, the generalizing capability of the proposed framework in real-world scenarios can be investigated. In the future, we may incorporate this by using a subject-independent evaluation mechanism for EEG signals. Furthermore, real-time feedback mechanisms, integration with facial expression, voice, and the use of explainable AI methods can further enhance results. It can improve the classification of human stress using psychological signals.

## Conclusion

In this proposed study, we have employed time-domain analysis for feature extraction to detect and classify human stress. These 20 temporal time-domain features are effectively used in the feature selection process. Out of these 20 features, 12 features have been emphasized in the classification process, as listed in Tables 2 and 3.

The performance of classifiers is evaluated using these 13 features by applying five machine learning methods, namely RF, DT, KNN, MLP, and ABM, as discussed in Sect. "Classification". Among these five classifiers, ABM and KNN have demonstrated the effectiveness of the proposed methodology for stress classification. In future work, the proposed framework will be evaluated using subject-independent validation strategies, such as leave-one-subject-out cross-validation, to assess its true cross-subject generalization capability.

## Data Availability

To support reproducibility and future research, the following resources have been made publicly available: The pre-processed EEG recordings from four electrodes, AF7, AF8, TP9, and TP10. The full set of extracted feature vectors and the computed engagement indices derived from these signals. All data are provided in CSV format and are available at: https://www.kaggle.com/datasets/usmanrauf/timedomain-segmentation-lemon.
